# Comparison of Pregabalin Versus Placebo in Reduction of Pain due to Lumber Disc Herniation

**DOI:** 10.7759/cureus.9985

**Published:** 2020-08-24

**Authors:** Deepak Kataria, Laraib Jumani, Muhammad Umer Ahmed, Vikash Kumar, Aamir Makda

**Affiliations:** 1 Internal Medicine, Shaheed Mohtarma Benazir Bhutto Medical University, Larkana, PAK; 2 Internal Medicine, Pakistan Institute of Medical Sciences, Islamabad, PAK; 3 Internal Medicine, Ziauddin University, Karachi, PAK; 4 Internal Medicine, Jinnah Sindh Medical University, Karachi, PAK; 5 Neurosurgery, Jinnah Postgraduate Medical Centre, Karachi, PAK

**Keywords:** lumber disc herniation, lower back pain, pregabalin

## Abstract

Introduction

Lower back pain is an extensive problem globally, and quite prevalent in Pakistan as well. In most cases of lower back pain, the cause is lumbar disc herniation. To treat this pain, there are various treatment options available. In this study, we aim to find the efficacy and safety of pregabalin in lower back pain due to lumbar disc herniation.

Methods

We conducted an open-label prospective trial in a public tertiary care hospital in Karachi, Pakistan, for a duration of five months, i.e. from July 2019 to December 2019. A total of 105 patients were randomized into two groups: pregabalin group and placebo group, and they were required to respond to visual analog scale (VAS) on day 0 and week 12 of the study.

Results

The results showed a significant reduction in pain over time in both the groups: pregabalin (p-value < 0.0001) and placebo (p-value < 0.0001). However, the difference in pain reduction between pregabalin and placebo was not significant (p-value = 0.57). The most commonly reported side effects were somnolence and dizziness.

Conclusion

Based on the results of this study, we conclude that adding pregabalin to non-steroidal anti-inflammatory drugs (NSAIDs) and physiotherapy had no significant effect on pain reduction. Further large-scale studies are needed to evaluate the role of gabapentoids in lower back pain due to lumbar disc herniation.

## Introduction

Lower back pain (LBP) is an extensive condition, with around 80% of the global population reporting such cases at least once in their lifetime [1]. In most scenarios, the principal cause reported is intervertebral degeneration initiating degenerative disc disease and lumbar disc herniation (LDH) [2]. Most LBP cases present with radiculitis, weakness in the lumbosacral plexus, and sensory anomalies [3]. Other reported symptoms include focal paresis, leg pain following coughing, heavy weight lifting and sneezing, and limited trunk flexion [3]. Around 40% of the known acute or chronic cases have reported constant pain during sitting posture due to increased disc pressure [4].

According to the National Institute for Health and Care Excellence (NICE) guidelines, most instances of back pain improve within a few weeks without any need of more potent analgesics like anticonvulsants [5]. There are some effectual non-opioid painkillers, and even Pilates has been found to be more efficacious [5,6]. However, many general practitioners still recommend and prescribe anticonvulsant medicines on a regular basis [7]. Although LBP is real and constant, these drugs restrict neuronal excitation and enhance inhibition [8]. It is not possible to indicate the effectiveness and safety of anticonvulsants, such as pregabalin in LDH and LBP, especially in Pakistan, due to uncertain statistics. Hence, this study aims to investigate the efficacy of pregabalin in patients suffering from LDH leading to persistent LBP.

## Materials and methods

We conducted an open-label prospective trial in a public tertiary care hospital in Karachi, Pakistan. The study duration was from July 2019 to December 2019.

During the study period, we included all patients of LDH, complaining of LBP. Both males and females were included in the study with ages between 18 and 70 years. Pregnant and lactating mothers were excluded from the study. The diagnosis of LDH was made radiologically by MRI. The severity of pain was evaluated using the visual analog scale (VAS). A score of 0 was equivalent to no pain and a score of 10 was recorded as the worst pain. Patients were required to respond to VAS on two instances, i.e. day 0 and week 12 of the study. Patients were asked to visit the clinic every four weeks to maintain follow-up where their general assessment was done, but the VAS score was not recorded. Age, gender, and VAS score were recorded in a self-structured questionnaire.

At the beginning of the study, we enrolled a total of 105 participants after taking informed consent. These participants were randomized into two groups: the pregabalin group (n = 52) and the placebo group (n = 53). The pregabalin group was prescribed 75 mg of pregabalin once a day with a non-steroidal anti-inflammatory drug (NSAID). In contrast, the placebo group was given only a NSAID. Participants of both groups were advised to undertake physiotherapy as well.

By the 4th week, no patient was lost to follow-up in either of the groups. However, by the 12th week follow-up, six participants were lost to follow-up in the pregabalin group and eight participants in the placebo group. Hence, at the end of the 12th week, the sample size reduced to 91. Out of these, 46 patients belonged to the pregabalin group and 45 patients belonged to the placebo group. Data were processed and analyzed using SPSS for Windows, version 22.0 (IBM Corp., Armonk, NY). Mean and standard deviation (SD) were calculated for continuous variables and frequencies, and percentages were calculated for categorical variables. For analysis of the mean scores of VAS, only those patients who completed the study (n = 91) were included, and for adverse events, all 105 patients were included. Mean was compared using the t-test. Adverse events were compared using the chi-square test. A p-value of less than 0.05 indicates that the difference between the pregabalin group and the placebo group is significant enough to discard the null hypothesis.

## Results

Among the 91 patients who completed the study, 41 were men (45%) and 50 were women (54.9%). Their mean age was 51 ± 8 years (range: 39-70 years).

Mean pain intensity recorded on VAS was compared for both pregabalin and placebo groups on day 0, and week 12. There was a significant reduction in pain over time within each group; however, the difference in pain reduction between the two groups was not significant (Table 1).

**Table 1:** VAS Score on Day 0 and Week 12 *Means of day 0 and week 12 within the groups were compared. **Means of week 12 for both groups were compared. SD: standard deviation; VAS: visual analog scale.

	Day 0 (n = 105)	Week 12 (n = 91)	P Value	
VAS			Intra Group*	Inter Group**
Pregabalin	6.89 ± 1.01	4.31 ± 0.56	<0.0001	0.57
Placebo	6.82 ± 0.93	4.38 ± 0.62	<0.0001	

The most common adverse event in pregabalin group was somnolence (19.2%), followed by dizziness (17.3%) (Table 2).

**Table 2:** Adverse Events Reported in Both Arms

Adverse Events	Pregabalin (n = 52)	Placebo (n = 53)	P Value
Somnolence	10 (19.2%)	1 (1.8%)	0.003
Dizziness	9 (17.3%)	1 (1.8%)	0.007
Fatigue	5 (9.6%)	1 (1.8%)	0.088
Weight gain	4 (7.6%)	1 (1.8%)	0.162
Edema	3 (5.7%)	0	0.248

## Discussion

Our study found that although pain reduced from day 0 to week 12 in both the groups (pregabalin and placebo), there was no significant difference in pain reduction between the pregabalin group and the placebo group. A meta-analysis and systemic review from 2018 echoes similar findings. They evaluated nine placebo-controlled randomized trials, similar to ours, for chronic LBP and lumbar radicular pain, and found high-quality evidence showing that gabapentinoids had no effect on pain and disability compared to placebo [9]. Another meta-analysis, published in PLOS Med, compared pregabalin to other analgesics and found a greater reduction in pain with other analgesics [10]. A similar recent review by Chou et al. on the role of anticonvulsants in LBP did not show any advantages [11]. These findings, including findings from our study, are different from the 2012 BMJ review, which reported treatment benefits of gabapentin based on a single trial [12].

The most common adverse event in the pregabalin group was somnolence, followed by dizziness and fatigue. Weight gain and edema were also numerically higher, but not statistically significant in the pregabalin group compared to the placebo group. Similar central nervous system adverse events were reported elsewhere as well. Dizziness was reported in 31% of cases with pregabalin, compared to 9% in the placebo group. Somnolence was experienced by 22% of patients treated with pregabalin compared to 7% of those receiving placebo [13]. Other possible adverse events affecting the central nervous system included visual blurring, asthenia, euphoria, gait imbalance, and cognitive difficulties. However, dizziness and somnolence are particularly significant side effects, as they can adversely impact driving skills and the operating of heavy pieces of machinery. 

There are various studies available in local literature that investigate the role of pregabalin in a post-surgical setting; however, to the best of our knowledge, this is the first study in our local setting that compares pregabalin vs placebo in LBP due to LDH. Nevertheless, this study has its limitations as well. It was a single-center study with small sample size; hence, care should be taken while inferring its data to a broader set of population.

In the UK, the prescription of anticonvulsants for back and neck pain, including radicular pain, by general practitioners, has increased by 535% in the last 10 years [14]. There is no such data available for prescription in Pakistan, but it can be said with certainty that there has been a significant increase in the prescription of anticonvulsants such as pregabalin for back and neck pain due to disc herniation. Our study plus various other meta-analyses have all shown that anticonvulsants such as pregabalin are ineffective in the treatment of chronic LBP and lumbar radicular pain. On the contrary, its use may be associated with increased risk of adverse events that may impact daily routine activities.

## Conclusions

In this study, pregabalin was found to be ineffective in significantly reducing LBP due to LDH, compared to placebo. Many other similar studies do not support the use of anticonvulsants for chronic LBP or lumbar radicular pain either. Consequently, we can state that pregabalin has notable adverse events, and clinicians should carefully consider the risks and benefits before prescribing it for patients with LBP.
